# Molecular and Evolution *In Silico* Studies Unlock the h4-HPPD C-Terminal Tail Gating Mechanism

**DOI:** 10.3390/biomedicines12061196

**Published:** 2024-05-28

**Authors:** Alfonso Trezza, Ancuta Birgauan, Michela Geminiani, Anna Visibelli, Annalisa Santucci

**Affiliations:** 1 Department of Biotechnology, Chemistry, and Pharmacy, University of Siena, Via Aldo Moro, 53100 Siena, SI, Italy; ancuta.birgauan@student.unisi.it (A.B.); geminiani2@unisi.it (M.G.); anna.visibelli2@unisi.it (A.V.); annalisa.santucci@unisi.it (A.S.); 2 SienabioACTIVE, Department of Biotechnology, Chemistry, and Pharmacy, University of Siena, Via Aldo Moro, 53100 Siena, SI, Italy; 3 ARTES 4.0, Viale Rinaldo Piaggio, 34, 56025 Pontedera, PI, Italy

**Keywords:** 4-HPPD, gating, mutagenesis, C-terminal tail, molecular modeling, molecular dynamics simulations, protein evolution, multiple sequence alignment

## Abstract

The enzyme 4-hydroxyphenylpyruvate dioxygenase (4-HPPD) is involved in the catabolism of the amino acid tyrosine in organisms such as bacteria, plants, and animals. It catalyzes the conversion of 4-hydroxyphenylpyruvate to a homogenisate in the presence of molecular oxygen and Fe(II) as a cofactor. This enzyme represents a key step in the biosynthesis of important compounds, and its activity deficiency leads to severe, rare autosomal recessive disorders, like tyrosinemia type III and hawkinsinuria, for which no cure is currently available. The 4-HPPD C-terminal tail plays a crucial role in the enzyme catalysis/gating mechanism, ensuring the integrity of the active site for catalysis through fine regulation of the C-terminal tail conformation. However, despite growing interest in the 4-HPPD catalytic mechanism and structure, the gating mechanism remains unclear. Furthermore, the absence of the whole 3D structure makes the bioinformatic approach the only possible study to define the enzyme structure/molecular mechanism. Here, wild-type 4-HPPD and its mutants were deeply dissected by applying a comprehensive bioinformatics/evolution study, and we showed for the first time the entire molecular mechanism and regulation of the enzyme gating process, proposing the full-length 3D structure of human 4-HPPD and two novel key residues involved in the 4-HPPD C-terminal tail conformational change.

## 1. Introduction

The enzyme 4-hydroxyphenylpyruvate dioxygenase (4-HPPD) is an Fe (II)-dependent, non-heme oxygenase that catalyzes the conversion of 4-hydroxyphenylpyruvate (4-HPP) to a homogenisate (HGA), the second step in the pathway for the catabolism of tyrosine [1]. The enzyme 4-HPPD is encoded by the HPD gene located on chromosome 12, represented by 14 exons and 4 transcripts (splice variants), 207 orthologues, and 1 paralogue associated with 6 phenotypes [1,2]. Almost all organisms express 4-HPPD in the cell cytoplasm (with the exception of some gram-positive bacteria), showing 393 residues with a 40–50 kDa subunit mass organized in a tetrameric (typically in bacteria) or dimeric form (human) [3]. Two domains characterize it; the vicinal oxygen chelate (VOC 1) domain (18–149 residues) and the VOC 2 domain (180–338 residues) (residue number based on the 4-HPPD with UniProt code P32754). The VOC domain is actually an enzyme family involved in catalyzing a wide variety of chemistries derived from a shared fundamental mechanism, which is the bidentate coordination to a divalent metal center (Fe (II)) by a substrate/intermediate/transition state with vicinal oxygen atoms [4]. The active site is located within a barrel-like β-sheet that accommodates a metal-binding site with an iron (II) ion as a cofactor. The iron ion is coordinated by a residue triad highly conserved, that is, H183, H266, and E349 [5].

The primary structure of the enzyme can be divided into a more variable N-terminus and a more conserved C-terminus, which folds into discrete domains. The active site is formed exclusively from residues of the C-terminus region; the function of the N-terminal half of the protein is not known [5,6].

The 4-HPPD C-terminal acts as a gate regulating the access to the active site and isolating the bound substrate during catalysis [7,8]. The coverage of the active site by a C-terminal extension is a recurring feature observed in numerous 2-oxoglutarate-dependent oxygenases [5]. The human 4-HPPD enzyme possesses a C-terminal tail that performs the regulatory function of gating, in contrast to the bacterial 4-HPPD enzymes found in organisms such as *Pseudomonas fluorescens* [9] or *Streptomyces avermitilis* [10], which show a C-terminal α-helix which regulates the enzyme gating mechanism [9,10,11]. 

E349 assumes a crucial role in catalysis, as it is proposed to be indispensable, both for the Fe(II)/substrate complex and for the activation of dioxygen upon substrate binding, representing the initial step in the 4-HPPD oxygenase activity. H266 plays a key role in the regulation of the geometry of the reactive oxygen intermediate (facilitating the coupling of decarboxylation and hydroxylation reactions). While H183 plays a critical role in metal ion coordination (determining the correct orientation of the reactive oxygen intermediate in the oxidative reaction), it represents an important step in protecting the protein from damage caused by alternative oxidative reactions. 

Structural, computational, and spectroscopic studies have shown that the binding of the substrate (HPP) triggers the activation of the metal center, leading to its susceptibility to binding the dioxygen [12,13,14,15].

The 4-HPPD catalysis process is allowed in the absence of water molecules inside the active site, and such a chemical condition is favored by F347, F371, P339, R378, and Q375 residues, which, following a rearrangement of the side chains, cause the coverage of the gate. Then, the water molecules are displaced, leading to the start of the substrate catalysis process. The C-terminal region plays a crucial role in the arrangement of residues surrounding the active site, regulating the gate opening/closing, and consequently, the start/end of the enzyme catalysis [6]. 

In the 4-HPPD 3D structure, the C-terminal N380, L381, T382, and N383 residues, forming an interaction network with K247, K248, E254, Y258, and Q375 (close to the active site), lead the C-terminal α-helix in the correct orientation for the gate regulation. Furthermore, the last 5 residues of the C-terminal (V389, V390, P391, G392, and M393) would seem to be key in the gate regulation. Unfortunately, no full-length 4-HPPD 3D structure is available to study the biological, functional, and structural role of the enzyme C-terminal tail. 

However, in vitro mutations of C-terminal residues highlighted important molecular insights about human 4-HPPD Q375 and R378 mutants (two residues belonging to the C-terminal tail) inducing a lack of the enzyme activity, showing the crucial role of this region for the enzyme biological function [6]. In-depth, the Q375N mutant causes the presence of the solvent in the active site, leading to the loss of enzyme function, while the mutation R378K causes the lack of the R378/E254 key structural interaction, showing its role in the regulation of the enzyme catalysis process [6].

Currently, the absence of the full-length 4-HPPD 3D structure does not allow the use of experimental methods to define the enzyme molecular mechanism, making the computational study the only useful approach to investigate the enzyme gating process.

To obtain biological, functional, and structural insights into human 4-HPPD, we applied an integrative and comprehensive bioinformatics pipeline to dissect and explore the dynamic features of the wild-type and experimental mutants to define the molecular mechanism responsible for enzyme gating regulation.

In this study, for the first time, novel key residues involved in enzyme gating were identified, providing fine regulation of the enzyme in/activation bringing to light important molecular fingers. Our results could open new frontiers in the target/drug discovery field, not only to further investigate human 4-HPPD-related diseases but also to propose new strategies to finely regulate the enzyme’s biological activity.

## 2. Methods

The 3D structure (PDB ID: 5EC3) and FASTA sequence (UniProtKB Entry: P32754) of the human 4-HPPD apo state were retrieved from the RCSB Protein Data Bank [16] and UniProt [17], respectively. The mutant 3D structures (Q375N, R378K, Y221A, and K39A) were obtained by the DUET tool [18]. To avoid errors during the molecular dynamic (MD) simulations, missing side chains and steric clashes in PDB files were adjusted through molecular modeling using PyMOD3.0 (Department of Biochemical Sciences, Sapienza University of Rome, Rome, Italy) [19] with MODELLER 10.5, and validated using PROCHECK [20]. CHARMM-GUI platforms [21] were used to prepare all systems for MDs using the charmm36-mar2019 force field to assign all molecular parameters, while GROMACS 2019.3 (University of Groningen Royal Institute of Technology Uppsala University, Uppsala, Sweden) [22] with CUDA support was used to perform the MD run. In brief, the structures were immersed in a cubic box filled with TIP3P water molecules and counter-ions to balance the net charge of the system. Simulations were run, applying periodic boundary conditions. The energy of the system was minimized, as suggested in previous work [23]. Thus, 5000 steps of minimization were set with the steepest descent algorithm to converge to a minimum energy with forces less than 100 kJ/mol/nm. All the cMD simulations were performed, integrating each time-step of 2 fs. A V-rescale thermostat maintained the temperature at 300 K, and a Noose–Hoover barostat maintained the system pressure at 1 atm with a low dumping of 1 ps^−1^. The LINCS algorithm constrained the bond lengths involving hydrogen atoms. The MD run was of 1µs for each system, and each MD was carried out 3 times (thus 15 MDs, for a total time of 15 µs). Next, a simulated annealing process to define the structural stability upon in silico mutagenesis was performed, setting six annealing times (from 0 ns to 5 ns) and six annealing temperatures (from 300 K to 550 K). All MD analyses were explored with GROMACS 2019.3 packages [22]. PCA analyses were performed for the entire trajectory of each system, considering the three triplicates, using the BIO3D implemented in Rstudio 4.1.2 (Posit, Vienna, Austria), while the free energy landscape was evaluated using the GROMACS 2019.3 package. Secondary structural analyses were carried out with Timeline, a VMD plugin.

GRACE [24] generated the MD graphs, and PyMOL 3.0 was used as a molecular graphic interface to produce the biological system pictures. To confirm the potential key residues, we applied an evolution approach using BLASTp 2.15.0 tool (National Library of Medicine, MD, USA) [25] to perform two different multiple sequence alignments (MSA) between the human 4-HPPD primary structure and (i) the “Non-redundant protein sequences (nr)” database, excluding the “*mammalia*” class, and (ii) the “Non-redundant protein sequences (nr)” database considering, only the “*mammalia*” class. The MSA was performed by using the BLOSUM 62 matrix, word size of 6, and max target sequences of 5000; all other parameters were used by default. The MSA results were analyzed and displayed through the Skylign 1.8.2 tool (HHMI Janelia Farm Research Campus, VA, USA) [26]. All data were computed using a workstation with CPU AMD Ryzen Threadripper PRO 5965WX 24 Core (4.5 GHz, 140 MB CACHE), DDR4 Kingston 3200 MHz 128 GB RAM, Nvidia GeForce RTX4090 24 GB graphic card, SSD NVME M.2 of 2 TB + 2x HDD S-ATA3 of 14 TB as storage partition with OS Ubuntu 14.04.

## 3. Results

### 3.1. Molecular Modeling

The 3D structure of human 4-HPPD is not yet entirely defined (376 modeled residues against 393 deposited residues); therefore, molecular modeling was applied to build and optimize its whole 3D structure. Firstly, the human 4-HPPD primary structure (target sequence) was extracted from the UniProt database (UniProtKB: P32754, 393 aa), and then, a multiple sequence alignment (MSA) with BLASTp was performed against the “Protein Data Bank” database. The MSA results revealed that the 3D structure “human 4-Hydroxyphenylpyruvate dioxygenase” (PDB code: 5EC3), solved by the X-ray diffraction method with a resolution of 2.10 Å, represented the best 3D structure to use as a template, showing identity and cover of 100% and 95.67%, respectively. To build the 3D structure of the target sequence using PyMOD 3.0, firstly, the 5EC3 sequence was aligned with the target sequence through Clustal Omega [27], and then the MODELLER 10.5 tool generated and optimized the whole target 3D structure. To obtain the best C-terminal tail conformation to the minimum energy, it was reconstructed through a “loop modeling” algorithm implemented in the MODELLER 10.5 tool, where the missing residues that belonged to the C-terminal region (378–393) were declared, thus a library of ten different tail conformations was provided, and the tail to the minimum energy was selected. The *Streptomyces avermitilis* 4-HPPD 3D structure (PDB ID: 1T47) was used to add the iron ion to our 3D model, performing a structural superimposition using the plugin “alignment” in PyMOL 3.0. The quality of the model was verified by Ramachandran plot (in PROCHECK 3.5.4 Tool) (Wellcome Genome Campus, Cambridge, UK) showing 100% of residues located in the most allowed regions, confirming the very high quality of the model. Finally, to relax the model, we solvated the system and performed, at first, an energy minimization (as described in the “methods” section); then, the system was led to 300 K and 1 bar, and a short MD run of 50 ns provided the relaxed model. Based on our 3D structure, the mutants Q375N, R378K, Y221A, and K39A were obtained using the DUET tool. The same protocols used to relax wild-type enzymes were applied to all 3D structures of the mutants.

### 3.2. Classical Molecular Dynamics Simulations (cMD)

#### 3.2.1. Root Mean Square Deviation (RMSD) Analyses

**Figure 1:**
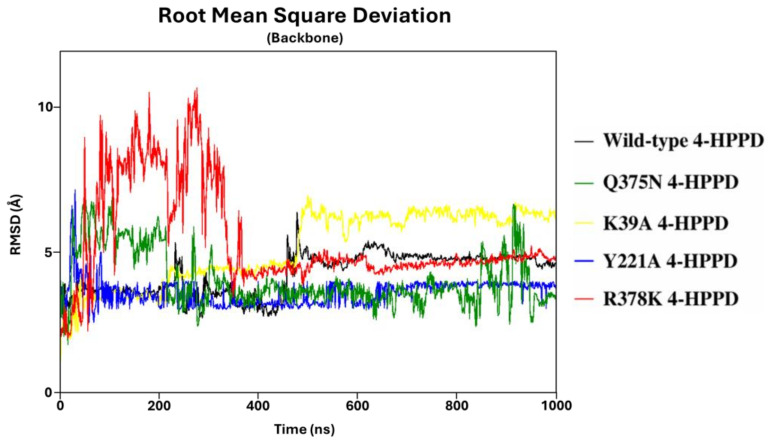
RMSD trends. RMSD profiles were evaluated for wild-type and mutant human 4-HPPD backbone along the entire MD run.

To define the structural stability of biological systems, the backbone RMSD of each structure was evaluated. From the backbone RMSD analysis, all systems achieved a structural stability of around 470ns (from 2 Å to 7 Å) (Figure 1). Interestingly, the Y221A, Q375N, and R378K mutants exhibited a lower RMSD value than the wild-type enzyme. RMSD profiles were also evaluated for all replicas for each system, considering the protein backbone and the protein backbone excluding the C-terminal tail, exhibiting a similar trend for each replica, indicating a good reproducibility of the MD run (Figure S1). The stable RMSD profiles showed the reliability of our simulations; thus, to identify potential key molecular insights involved in the 4-HPPD gating function, all biological systems underwent further analyses.

#### 3.2.2. Hydrogen Bond Network and Conformational Analyses

To identify potential pivotal residues involved in the 4-HPPD gating regulation, the interaction network between the C-terminal tail residues (378–393) and the residues of the whole protein surface was explored for each system (considering the three replicates) creating an extra index with the “gmx make_ndx” function in GROMACS 2019.3. 

Surprisingly, the wild-type 4-HPPD interaction network results showed the formation of a hydrogen bond (evaluated through the “existence map” with the “gmx hbond” function in GROMACS 2019.3) between the Y221-R378 and K39-M393 residues with a notable occupancy of around 74.60% and 51.20%, respectively, considering the MD run time from the first contact (Figure S2 and Movies S1 and S2).

Remarkably, no hydrogen bond with Y221 and K39 was detected for all mutants in each replica.

To dissect the 4-HPPD gating regulation, our study identified and compared the conformational differences between wild-type 4-HPPD and its mutants, considering the C-terminal tail residues. 

Conformational analyses were conducted for each system (for the three replicates) based on the interaction network analyses previously described, and then, by using the PyMOL 3.0 3D molecular graphical interface software, all MD trajectories were analyzed. 

**Figure 2:**
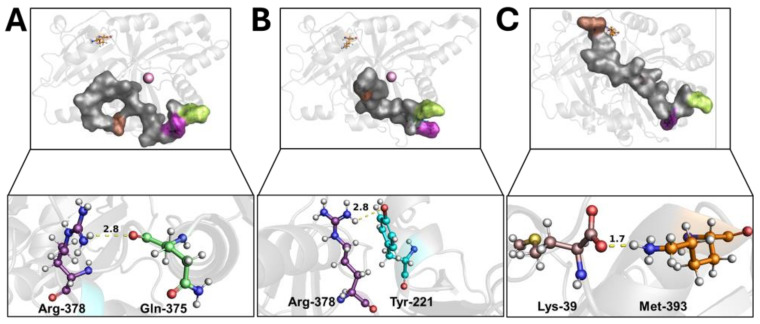
C-terminal tail shaping process. The h4-HPPD is reported as a transparent gray cartoon, while the C-tail is presented as a transparent gray surface. The residues involved in hydrogen bond formation are reported in orange (Lys-39), cyan (Tyr-221), green (Gln-375), purple (Arg-378), and brown (Met-393) ball-and-stick. The iron ion is represented as a pink sphere. The hydrogen bond is shown as a yellow dotted line, the number on the hydrogen bond indicates the length of the H-bond. (**A**) hydrogen bond between the Gln-375 and Arg-378, (**B**) hydrogen bond between the Tyr-221 and Arg-378, (**C**) hydrogen bond between the Lys39 and Met-393.

Wild-type 4-HPPD exhibited a pre-equilibrium phase for the initial 400 ns; at this time, despite the tail’s super-flexible behavior, a hydrogen bond between the Q375 and R378 residues was formed (Figure 2A). From 400 ns to 500 ns, a weak hydrogen bond between R378 and Y221 was trigged (lacking the Q375-R378 hydrogen bond). From 500 ns, the formation of a stable hydrogen bond (for the whole MD run) between Y221 and R378 was observed (Figure 2B) when the C-terminal loop started a shaping process, and, following a significant structural torsion, the C-terminal tail was oriented towards the N-terminal region, allowing the formation of a strong hydrogen bond between the M393 and K39 residues (Figure 2C), achieving a C-terminal tail conformation able to wholly cover the enzyme active site, causing a “close conformation” (active state). Following the lack of a K39-M393 hydrogen bond, the C-terminal tail uncovered the active site, leading the enzyme into an “open conformation” (inactive state) (Movie S3). 

**Figure 3:**
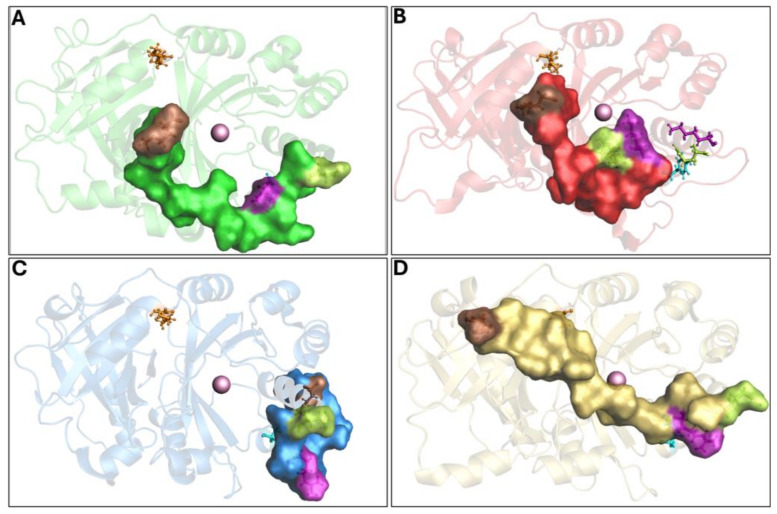
Overview of 4-HPPD inactive state. The 4-HPPD is reported as a green (Q375N), red (R378K), blue (Y221A), and yellow (K39A) transparency cartoon, respectively, while the C-terminal tail is reported as a surface. Arg-378 (purple), Gln-375 (green), Tyr-221 (cyan), Met-393 (brown), and Lys-39 (orange) are reported in ball-and-stick format. The Fe(II) ion is represented as a pink sphere. (**A**–**C**) For the Q375N (green), R378K (red), and Y221A (blue) mutants a 4-HPPD inactive state for the entire MD run is shown, where the enzyme active site shows being fully solvent accessible. (**D**) K39A mutant (yellow) shows a C-terminal tail conformation, which partially covers the enzyme active site, leading the 4-HPPD into a “semi-open” conformation.

The Q375N mutant showed a very high flexibility in the C-terminal tail movement, not allowing the formation of the hydrogen bonds observed in wild-type 4-HPPD, causing an incorrect orientation of the C-terminal tail, leading to the enzyme being in an “open conformation” (inactive state) (Figure 3A and Movie S4). 

The R378K mutant achieved an equilibrium state around 400 ns, and no hydrogen bond was observed with Y221. As a result of the cMD run, the C-terminal tail was not able to achieve the correct orientation, like the wild-type, leading the enzyme to an “open” conformation (inactive state) (Figure 3B and Movie S5). 

Q375N and R378K mutant would suggest a role of Y221 and K39 in the enzyme gating regulation, to validate our hypothesis, Y221A and K39A mutants were generated in silico, and conformational analyses were performed by applying cMD. 

The Y221A mutant showed the initial hydrogen bond between Q375 and R378 in the first 100 ns; however, during the entire cMD run, the C-terminal tail was not able to achieve a similar shape to the wild-type; rather, its conformation was comparable to the “open” conformation (inactive state) of the Q375N and R378K mutants (Figure 3C and Movie S6).

The K39A mutant showed a similar interaction network to that reported for the wild-type enzyme, forming, firstly, a hydrogen bond between R378 and Q375 and then a hydrogen bond between Y221 and R378, Following such interaction, the C-terminal tail was oriented toward the N-terminal region; here, the mutation K39A did not allow the hydrogen bond with M393, leading the conformation into a “semi-open” (semi-active state) state (Figure 3D and Movie S7). 

#### 3.2.3. Principal Component Analyses (PCAs) and Free-Energy Landscape (FEL) 

**Figure 4:**
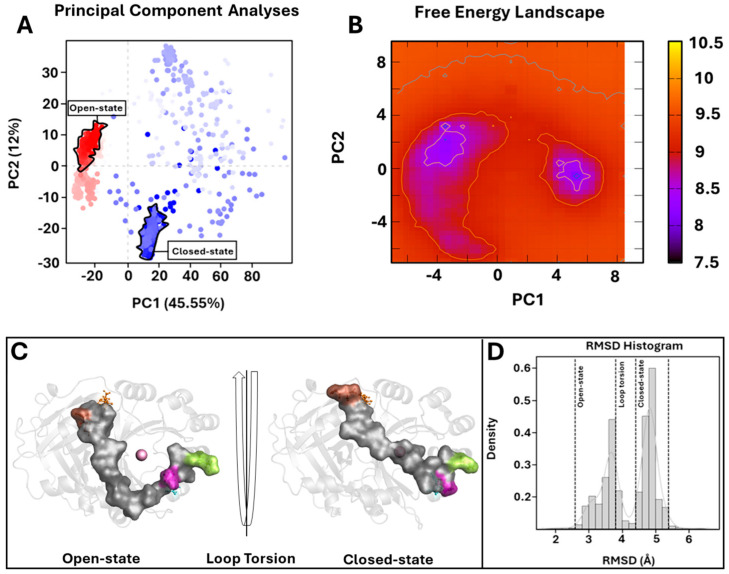
PCA overview. (**A**) For the PCA, 1000 equidistance conformations were analyzed for the entire MD run (1000 ns). PC1 and PC2 were plotted against each other using the backbone carbon atoms. (**B**) The FEL values were constructed as a function of projections of the MD trajectory onto their own first (PC1) and second (PC2) eigenvectors, respectively. The color bar represents the relative free-energy value in kcal/mol. (**C**) Overview of 4-HPPD in/active state. The 4-HPPD is reported as a gray transparent cartoon, while the C-terminal tail is reported as a gray surface. Arg-378 (purple), Gln-375 (green), Tyr-221 (cyan), Met-393 (brown), and Lys-39 (orange) are reported in ball-and-stick format. The Fe(II) ion is represented as a pink sphere, showing the 4-HPPD active (the enzyme active site is fully solvent accessible) and inactive states (the C-terminal tail conformation wholly covers the enzyme active site). (**D**) RMSD distribution along the entire MD run.

To further investigate the enzyme conformational changes and their different states, PCA and FEL studies were performed. PCA analyses of MD trajectories of each system (considering the three replicates) revealed that only the wild-type 4-HPPD eigenvectors possessed significant eigenvalues (Figures S3–S5). In particular, the first 3 eigenvectors represented the 67.4% (first replica), 84.8% (second replica), and 67.7% (third replica), of the entire conformational population. Therefore, most of the conformations of wild-type 4-HPPD were confined within a subspace of very small dimensions (Figure 4A), equally for the replicates (Figures S6A and S7A), representing mainly the open and closed states. PCA analyses obtained for each mutant in each replica never showed eigenvectors with a significant eigenvalue, exhibiting only one main conformational state (Figure S8A–D). 

To further support the PCA evidence, the FEL evaluation was performed for each system (considering each replica). The FEL values of the wild-type 4-HPPD were constructed using the projections of its own first (PC1) and second (PC2) eigenvectors, given as CV1 and CV2, respectively, in the MD simulation. For the wild-type enzyme, there were two main free-energy wells in the global free-energy minimum region (Figure 4B), as well as for each replica (Figures S6B and S7B), showing two stable conformational states for the enzyme (open and closed). Remarkably, for each mutant in each replica, there was only one main free-energy basin in the global free-energy minimum region, indicating only one stable conformational state located within this well (Figure S9A–D). FEL results strongly confirmed the PCA evidence, suggesting two main stable conformational states, open and closed (Figure 4C) (Figures S6C and S7C). RMSD distribution analyses, performed for each system considering each replica, showed a clear presence of two main conformational states for the wild-type enzyme (Figure 4D and Figures S6D and S7D).

#### 3.2.4. Multiple Sequence Alignment (MSA)

The role of Y221 and K39 in gating regulation was further explored, applying an evolutionary approach, to observe the conservation of residues along the enzyme evolution. The BLASTp tool performed MSA between the human 4-HPPD primary structure and (i) the “non-redundant protein sequences (nr)” database excluding the “*mammalia*” class, and (ii) the “non-redundant protein sequences (nr)” database considering only the “*mammalia*” class. 

**Figure 5:**
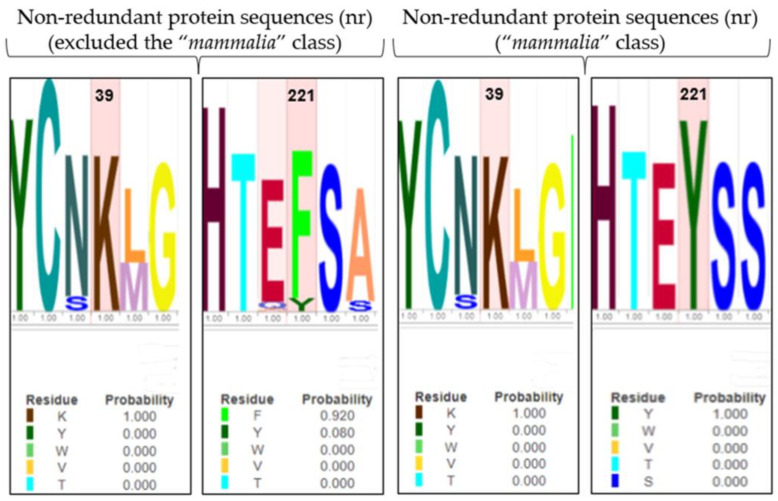
LOGO representation of MSA of the 4-HPPD primary structure for all organisms (except the “*mammalia*” class), and for only the “*mammalian*” class. The height of the stack typically corresponds to the conservation at that position, and the height of each letter (amino acid) within a stack depends on the frequency of that letter at that position. A unique color is assigned to each amino acid. The x-axis indicates the mutation frequency (a value of 1 indicates the maximum conservation, value of 0 indicates no conservation) of the residue along the evolution.

Y221 was poorly conserved in all organisms (excluding the “*mammalia*” class) indicating an amino acid change in phenylalanine, with a mutation frequency of 92%. Differently, K39 was always conserved (Figure 5). Surprisingly, in “*mammalia*” class, Y221 exhibited perfect conservation during the enzyme evolution, as well as K39. 

#### 3.2.5. Simulated Annealing (SA) 

**Figure 6:**
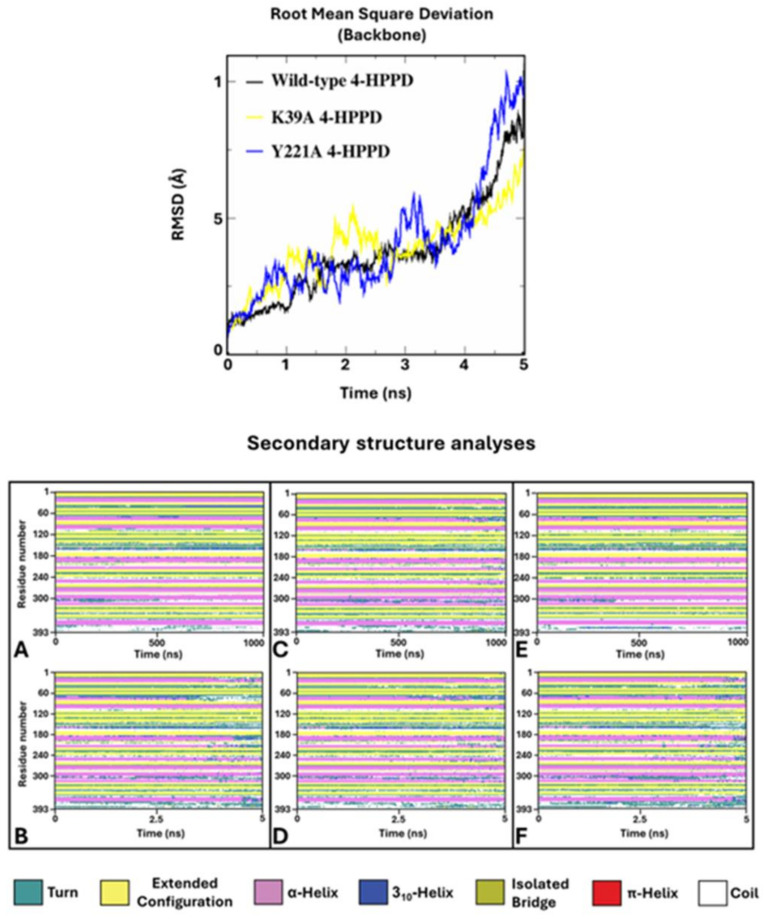
SA-MD RMSD profile and secondary structure analyses. In the top panel, the h4-HPPD backbone RMSD trends along SA-MD. The bottom panel shows the comparison of the secondary structure analyses between the cMD (**A**) is wild-type, (**C**) is K39A, and (**E**) is Y221A and SA-MD (**B**), (**D**), and (**F**) panels represent the wild-type, K39A, and Y221A, respectively.

To verify potential enzyme structural destabilizing effects upon the mutations Y221A and K39A, we performed SA-MD to observe the structural stability of systems and compare them with the wild-type 4-HPPD structural stability. Each biological system was prepared using the same protocols used in cMD; differently, MD run protocols were modified according to the SA-MD protocol. In brief, the biological system was thermos-equilibrated to 300 K, then gradually heated to 550 K in a six-time-step scale. SA-MD results were evaluated by computing the backbone RMSD values of each system. RMSD analyses provided a very similar trend among the mutants compared to wild-type enzymes. In detail, the 3D structures showed good structural stability from 0 ns (300 K) to 3 ns (450 K). Then, a rapid growth of RMSD values was observed for each system. SA-MD finished with a backbone RMSD value from 7.5 Å to 10 Å, comparable to the wild-type enzyme RMSD trend (Figure 6).

To define the potential protein misfolding, a secondary structure analysis was performed for the wild-type and mutant 4-HPPD along the entire MD run. Secondary structure analyses clearly showed a misfolding process of the enzyme for each system, confirming similar structural stability of the mutants to the wild-type enzyme. 

## 4. Discussion

Human 4-HPPD catalyzes the conversion of 4-hydroxyphenyl pyruvic acid to homogentisic acid, one of the steps in tyrosine catabolism. 

The catalysis process occurs within the active site and several types of reactions are catalyzed by these oxygenases, including hydroxylations, desaturations, and oxidative ring closures, and the reaction environment may have pharmaceutical and medical implications. The conversion of substrate is very complex, involving oxidative decarboxylation, side-chain migration, and aromatic hydroxylation in a single catalytic cycle. 

A barrel-like β-sheet buries the human 4-HPPD active site, which is finely regulated by a C-terminal tail able to cover the active site, assuming a gate function regulating the access of the substrate to the active site and isolating the bound substrate during catalysis.

Human 4-HPPD alterations have been observed in severe and rare diseases like tyrosinemia type III and hawkinsinuria, for which no cure is available. Hence, knowing the 4-HPPD gating molecular mechanism represents a crucial step in regulating and/or using 4-HPPD as a therapeutical target. 

In vitro experiments showed the key role of the C-terminal tail for enzyme activity through truncation experiments. 

Here, applying an integrative bioinformatics/evolution study, wild-type and mutant 4-HPPD were deeply dissected and explored, bringing to light for the first time an enzyme-gating molecular regulation mechanism, providing the human 4-HPPD full-length 3D structure, and revealing Y221 and K39 as two novel pivotal amino acidic residues that steer and stabilize the C-terminal tail conformational change defining the enzyme in/activation.

RMSD analyses confirmed the structural stability of systems considered in this study, and the reliability of the MD protocols. Furthermore, considering the protein backbone of RMSD without the C-terminal tail, a significantly lower and stable RMSD profile was obtained, and such evidence would show the super-flexible behavior of the C-tail within the protein structure. 

Based on the experimental data, which showed the role of the C-terminal tail in the gating mechanism, we identified potential residues involved in this process by considering significant interactions between the C-terminal tail residues and any other residue present on the protein surface. Interaction network analyses performed for wild-type and Q375N and R378K mutant 4-HPPD provided interesting results. 

Wild-type 4-HPPD exhibited the formation of hydrogen bonds for Y221-R378 and K39-M393 residues with significant occupancy, indirectly showing a close distance between the residues; differently, no hydrogen bond with Y221 and K39 was detected for all mutants, suggesting their potential involvement in the gating regulation.

PCA analyses showed, with high statistical accuracy, two different conformational states only for the wild-type 4-HPPD, representing the open and closed states following the conformational change of the C-terminal tail, and to further support our results, the FEL of each system was explored. Wild-type 4-HPPD showed two energy wells, characterized by a larger, more “rugged” and complex free-energy surface than all mutants. Such evidence would confirm that the wild-type enzyme had two conformational sub-states (open and closed), richer than conformational diversity, and more complex dynamics behavior than all other mutants. The RMSD distribution further validates our results, clearly providing two different conformational states of the wild-type enzyme throughout the entire MD simulation. Despite the three replicates, the wild-type enzyme showed a similar result for all the analyses performed; a slight difference was noted for the PCA and FEL. Such a difference could be explained by the super-flexible behavior of the C-terminal tail. Since it is made up of 19 residues, its behavior can slightly change both in the time of formation of interactions and in the movement to reach the N-terminal region; however, the wild-type enzyme, in the three triplicates, is able to always reach an open and closed state following the conformational change of the C-terminal loop. Instead, differently from the wild-type enzyme, all mutants never showed a significant PCA result or two stable states, based on the FEL, strongly supporting our analyses. 

Conformational analyses carried out for the wild-type enzyme showed the Q375-R378 hydrogen bond formation. Such an interaction would lead to a first stabilization of the C-terminal tail, allowing the formation of a second hydrogen bond between R378 and Y221. Here, Y221 steers the C-terminal tail toward the N-terminal region (open/inactive state). Finally, the K39-M393 hydrogen bond would stabilize the C-terminal tail in a conformation able to wholly cover the enzyme active site (close/active state). 

Our results would suggest the hypothesis that the absence of the initial interaction between Q375 and R378 may influence the C-terminal tail stabilization, avoiding hydrogen bond formation with Y221 and C-tail movement toward the N-terminal region.

The conformational analyses of Q375N and R378K mutant 4-HPPD confirmed our hypothesis; in fact, no hydrogen bond with Y221 was detected, causing a high flexibility and random conformations of the C-terminal tail, avoiding its correct shaping toward the N-terminal region, leading the enzyme to be in an open/inactive state for the whole MD run. 

Such evidence would suggest the effective potential role of the Y221 and K39 residues in the enzyme gating regulation; therefore, Y221A and K39A mutants were obtained in silico to evaluate their dynamics behaviors.

The Y221A mutant analyses showed, firstly, the Q375-R378 hydrogen bond formation, and then the movement of the C-terminal tail toward A221, where no hydrogen bond was observed, leading to the conformation of the C-terminal tail in a shape like that of the Q375N and R378K mutants, confirming the potential role of Y221 in enzyme gating.

The K39A mutant provided significant evidence to confirm our study, where, firstly, the hydrogen bond between R378 and Q375 was established and subsequently the Y221-R378 hydrogen bond occurred; thus, the C-terminal tail was oriented toward the N-terminal region. Here, the presence of A39 did not allow the hydrogen bond formation with M393, leading to a destabilization of the C-terminal tail, causing a “semi-open/semi-active” conformation. Our result would be heavily supported by experimental evidence that 4-HPPD is involved in hawkinsinuria disease as a result of an alanine-to-threonine mutation at position 33 in the human enzyme [28], causing an incorrect lock of the active site, which causes the entrance of water, leading to the formation of catalytic aberrations. Interestingly, A33 is located on the same α-helix of K39, and the mutation of A33T could determine a potential structural distortion of the α-helix or a potential polar interaction with M393 during its movement toward K39, not allowing the correct shape of the C-terminal tail, causing the “semi-open/semi-active” conformation of the enzyme.

The evolution approach (MSA analyses) showed that Y221 and K39 were always conserved during the protein evolution in the “*mammalia*” class, while in all other organisms (excluding the “*mammalia*” class), Y221 was poorly conserved with a substitution in phenylalanine.

On first consideration, this evidence would disprove our result; actually, the C-terminal tail (involved in the gating mechanism) has been acquired only in the “*mammalia*” class; interestingly, Y221 in all other organisms was present as phenylalanine, representing a conservative mutation, suggesting a potential functional/structural role; thus, it is highly conceivable that there is a co-evolutionary process between Y221-R378 and K39-M393 to establish the hydrogen bond pattern to perform the role of fine regulators of the enzyme gating mechanism.

Annealing molecular dynamics and secondary structural analyses showed the structural stability of Y221A and K39A upon mutation, providing a similar RMSD trend compared to wild-type 4-HPPD, indicating no structural destabilization due to the mutation proposed by us. 

## 5. Conclusions

In vitro evidence showed the key role of the C-terminal tail for enzyme activity through truncation experiments; unfortunately, the molecular basis of the C-terminal tail in the enzyme gating mechanism remains unknown due to the absence of the full-length 4-HPPD 3D structure, making the computational approach the only possible method to explore the enzyme molecular insights. 

In this study, we showed how, in human 4-HPPD activity, the first interaction between Q375 and R378 would allow the Y221-R378 hydrogen bond formation, causing a first stabilization of the C-terminal tail, with Y221 acting as a pivot orienting the C-terminal tail toward the N-terminal region (open/inactive state), where the K39-M393 hydrogen bond would stabilize the C-terminal tail conformation to wholly cover the enzyme active site (close/active state). 

Taken together, our results show the fine regulation, steered by Y221 and K39, of the C-terminal tail conformation in the human 4-HPPD gating mechanism, providing important molecular insights useful to open and explore new frontiers in the target discovery field to propose innovative therapeutical approaches for human 4-HPPD-related rare diseases and disorders and precision medicine interventions. 

## Data Availability

The original contributions presented in the study are included in the article/Supplementary Material, further inquiries can be directed to the corresponding author.

## Supplementary Materials

The following supporting information can be downloaded at: https://www.mdpi.com/article/10.3390/biomedicines12061196/s1. Figure S1. RMSD profiles. Figure S2. H-bond network. Figure S3. PCA (Replica 1). Figure S4. PCA (Replica 2). Figure S5. PCA (Replica 3). Figure S6. PCA overview (Replica 2). Figure S7. PCA overview (Replica 3). Figure S8. PCA overview of mutant 4-HPPD. Figure S9. FEL values of mutant h4-HPPD for each replica. Movie S1. Y221-R378 H-bond network. Movie S2. K39-M393 H-bond network. Movie S3. Wild-type 4-HPPD MDs. Movie S4. Q375N 4-HPPD MDs. Movie S5. R378K 4-HPPD MDs. Movie S6. Y221A 4-HPPD MDs. Movie S7. K39A 4-HPPD MDs.
