# Supplementary figures and images for: Molecular and Evolution In Silico Studies Unlock the h4-HPPD C-Terminal Tail Gating Mechanism
Source: Biomedicines. 2024 May 28;12(6):1196. doi: 10.3390/biomedicines12061196 (PMC11201076; doi:10.3390/biomedicines12061196)

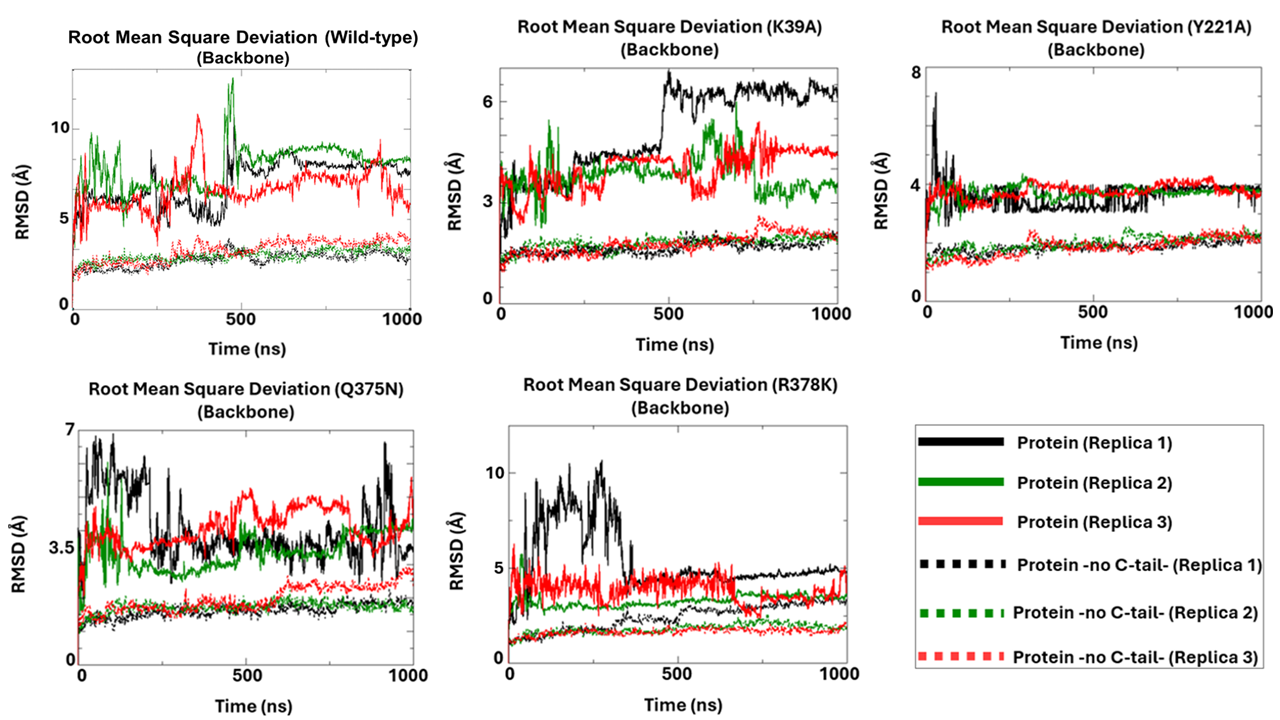

Supplement: Supplementary file 1 [file biomedicines-12-01196-s001.zip › Supplementary Materials/Figure S1.PNG]

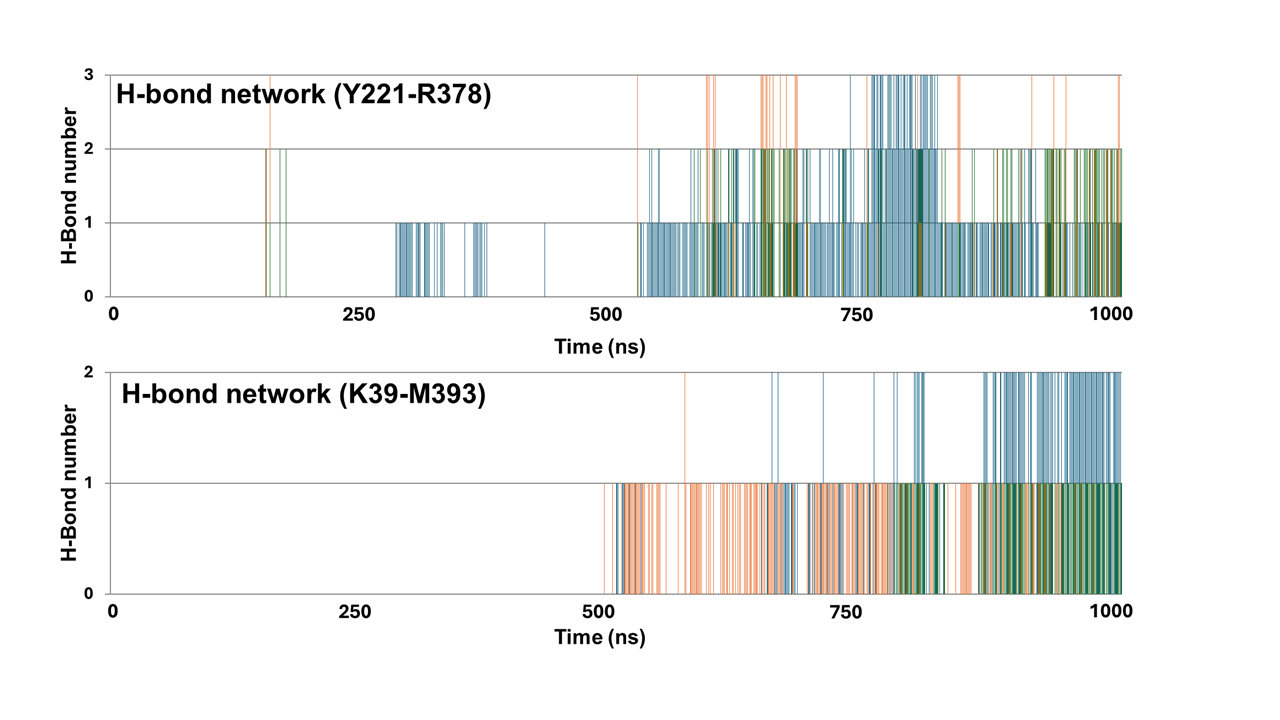

Supplement: Supplementary file 1 [file biomedicines-12-01196-s001.zip › Supplementary Materials/Figure S2.PNG]

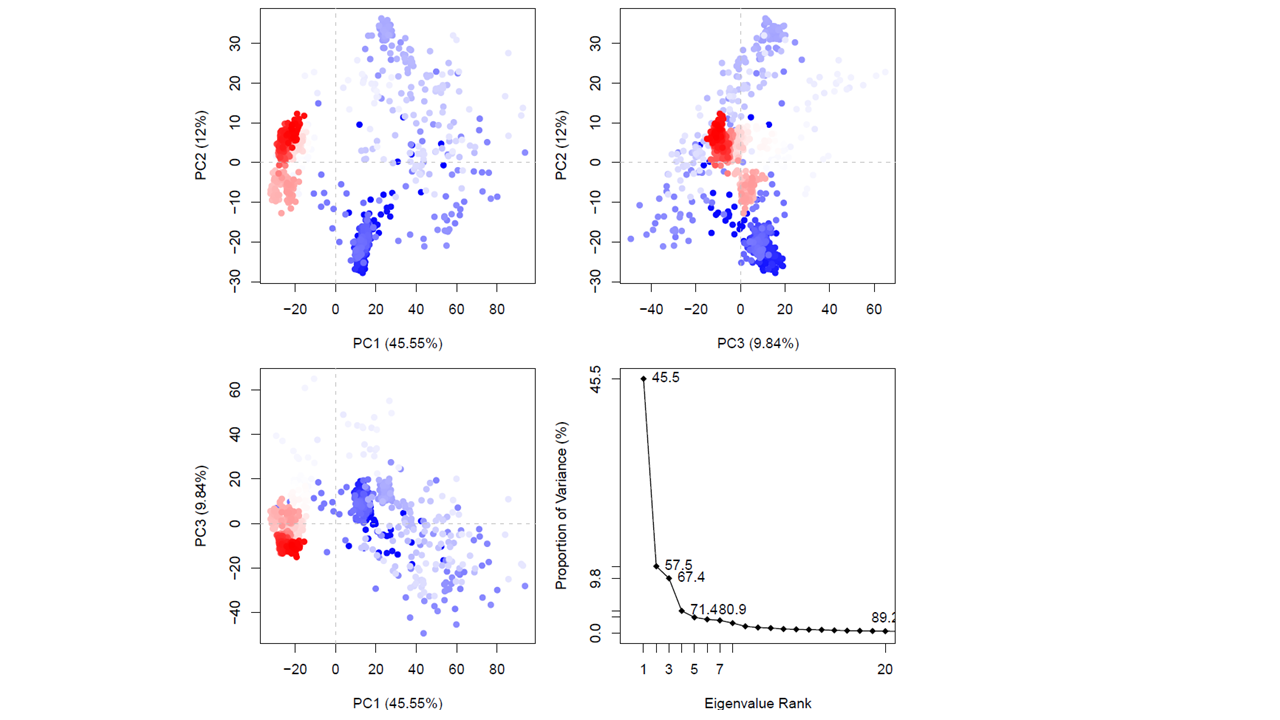

Supplement: Supplementary file 1 [file biomedicines-12-01196-s001.zip › Supplementary Materials/Figure S3.PNG]

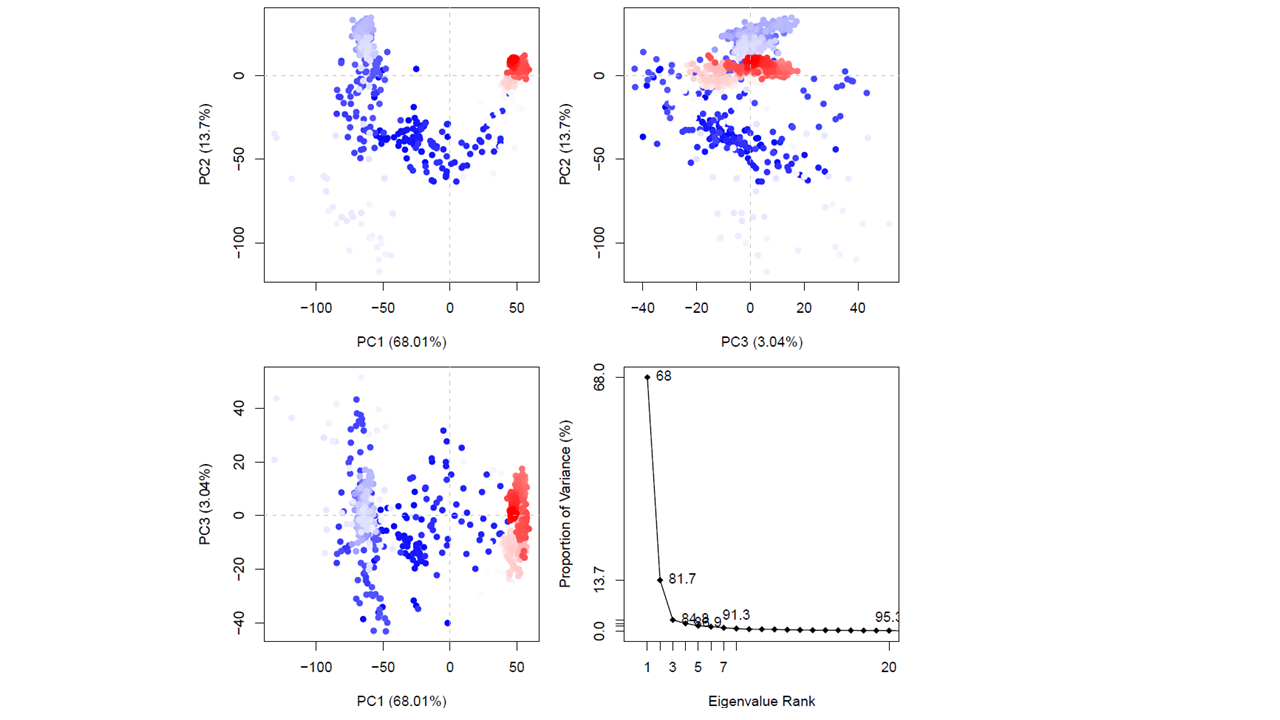

Supplement: Supplementary file 1 [file biomedicines-12-01196-s001.zip › Supplementary Materials/Figure S4.PNG]

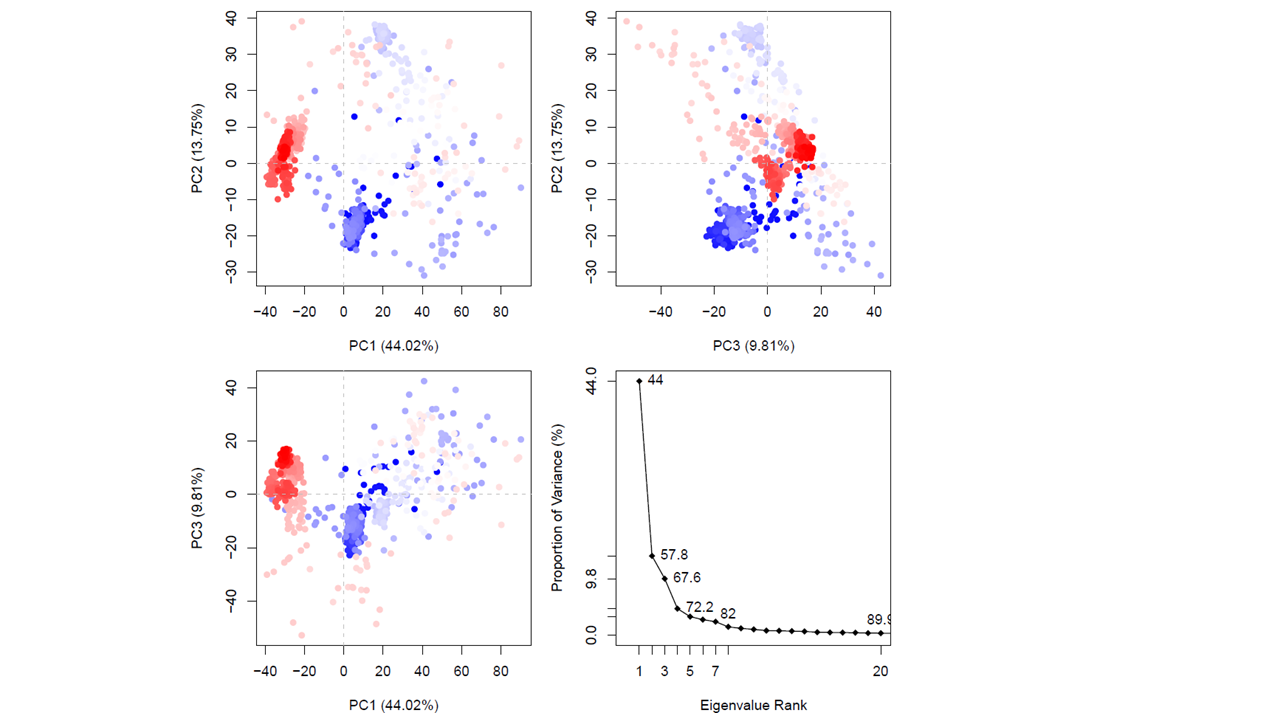

Supplement: Supplementary file 1 [file biomedicines-12-01196-s001.zip › Supplementary Materials/Figure S5.PNG]

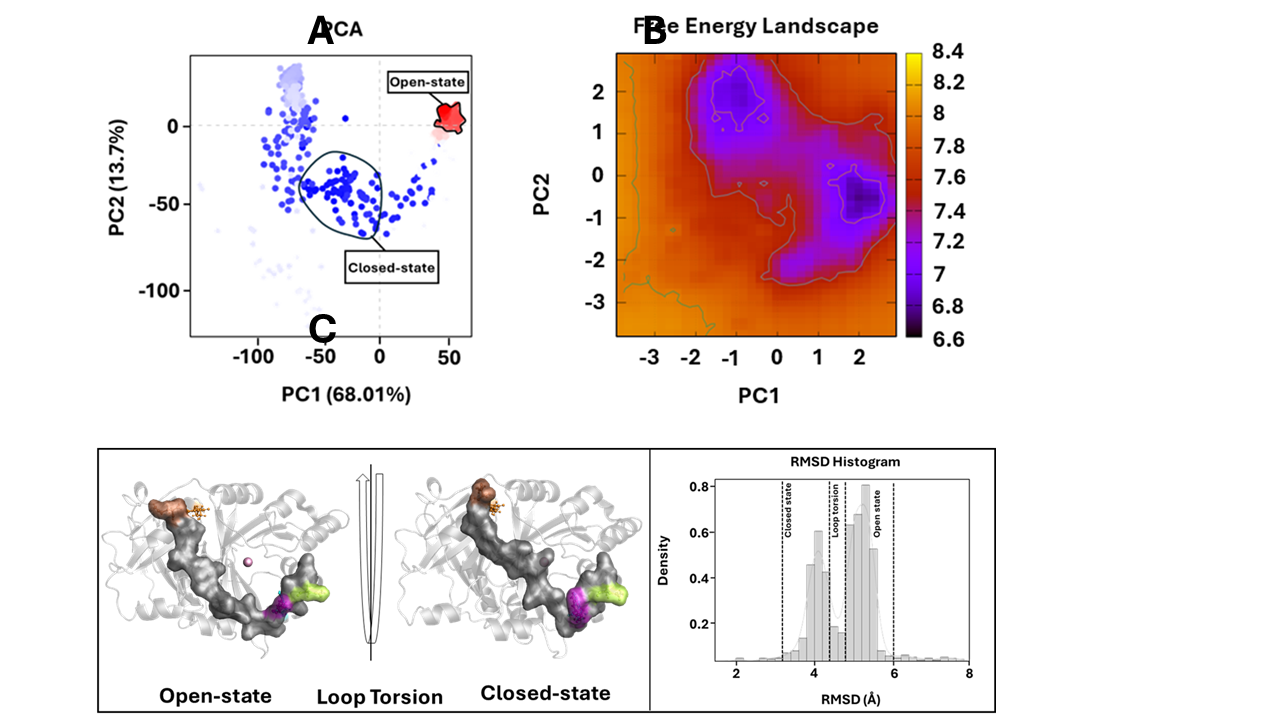

Supplement: Supplementary file 1 [file biomedicines-12-01196-s001.zip › Supplementary Materials/Figure S6.PNG]

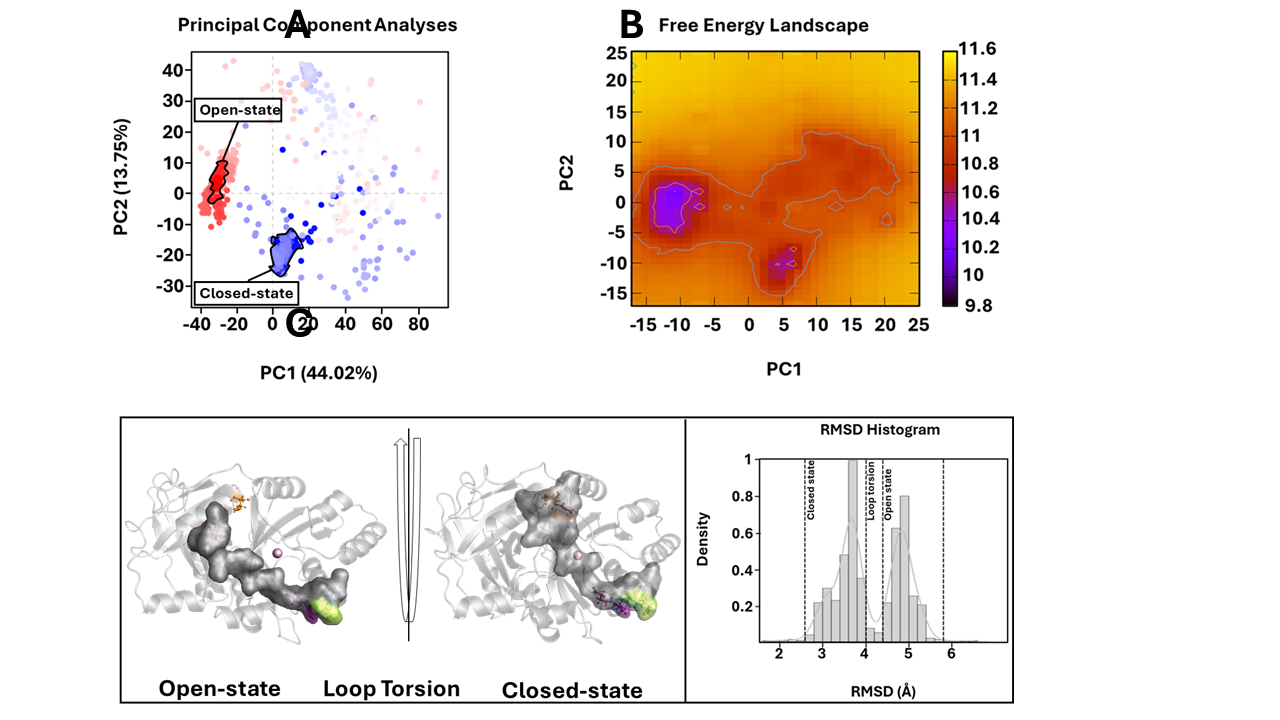

Supplement: Supplementary file 1 [file biomedicines-12-01196-s001.zip › Supplementary Materials/Figure S7.PNG]

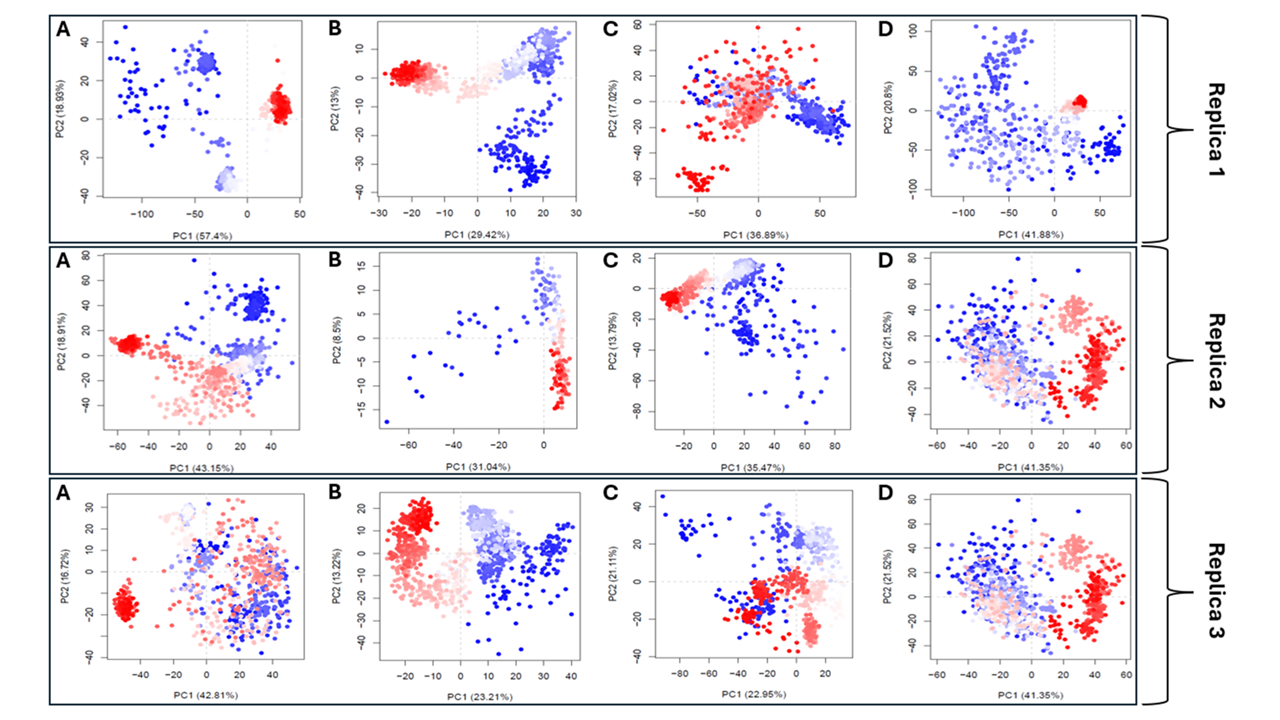

Supplement: Supplementary file 1 [file biomedicines-12-01196-s001.zip › Supplementary Materials/Figure S8.PNG]

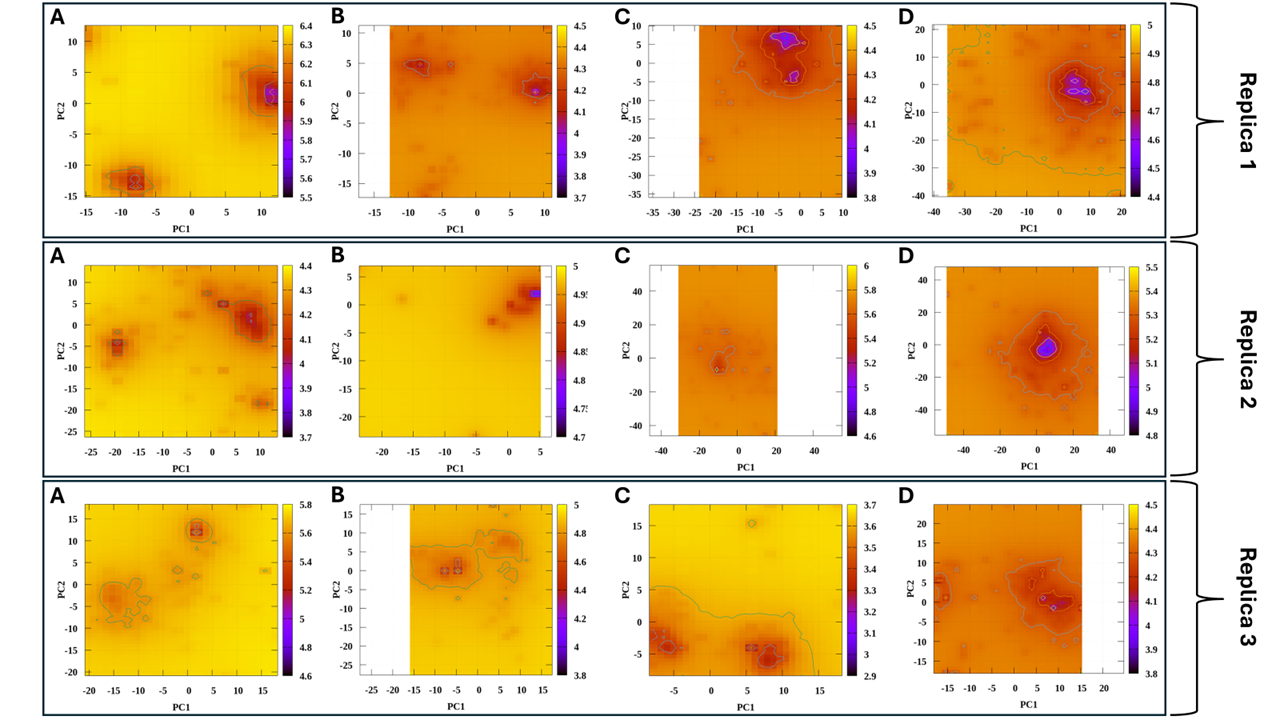

Supplement: Supplementary file 1 [file biomedicines-12-01196-s001.zip › Supplementary Materials/Figure S9.PNG]
